# Analysis of glucocerebrosidase (GBA) gene mutations in Iranian patients with Gaucher disease

**DOI:** 10.22037/ijcn.v15i4.23834

**Published:** 2021

**Authors:** Hadi MOZAFARI, Mohammad TGHIKHANI, Zohreh RAHIMI, Asad VAISI RAYGANI, Shahla ANSARI, Shohreh KHATAMI, Mohammad Reza ALAEI, Reza SAGHIRI

**Affiliations:** 1 Medical Biology Research Center, Kermanshah University of Medical Sciences, Kermanshah, Iran; 2 Department of Clinical Biochemistry, Faculty of Medical Sciences, Tarbiat Modares University, Tehran, Iran; 3 Department of Clinical Biochemistry, Medical School, Kermanshah University of Medical Sciences, Kermanshah, Iran; 4 Department of Hematology and Oncology, Faculty of Medicine, Iran University of Medical Sciences, Tehran, Iran; 5 Department of Biochemistry, Pasteur Institute of Iran, Tehran, Iran; 6 Department of Pediatrics, Faculty of Medicine, Shahid Beheshti University of Medical Sciences, Tehran, Iran

**Keywords:** Gaucher Disease, Mutation, GBA, Sequencing

## Abstract

**Objectives:**

Gaucher disease (GD) is the most common autosomal recessive disorder of glycolipid storage. It results from mutations in the glucocerebrosidase (*GBA*) gene and leads to GBA deficiency. Different mutations are associated with different phenotypes in the three major types of GD.

**Materials and Methods:**

The spectrum of mutations in *GBA* gene in 26 unrelated patients with GD from different Iranian populations was determined by DNA sequencing, polymerase chain reaction (PCR)-restriction fragment length polymorphism (RFLP), and amplification-refractory mutation system (ARMS) methods. An *in silico* analysis was also performed for novel mutations.

**Results:**

Six new mutations were identified in this study. The newly detected mutations that could be theoretically harmful included p.I200T (c.599T>C), p.H312D (c.934C>G), p.L325S (c.974T>C), p.L393V (c.1177C>G), p.S439G (c.1315A>G), and p.M455R (c.1365G>A). Also, p.L483P, p.N409S, p.W420X, p.E379K, p.R398Q, p.N227S, p.R202Q, and p.D448H mutations were identified in the patients. Besides, two new complex mutations, namely, p.S439G/p.S439G+p.E379K/- and p.R202Q/p.R202Q+p.N227S/p.N227S, were detected. The most common *GBA* mutation in the population was p.L483P with an allele frequency of 32.7%, followed by p.N409S (19.2%).

**Conclusion:**

The present study detected six new mutations of *GBA* gene among GD patients. Two mutations (p.L483P and p.N409S) were especially common among Iranians; this finding can be used in implementing screening programs and understanding the molecular basis of GD.

## Introduction

Gaucher disease (GD) is an autosomal recessive disorder and the most common form of lysosomal storage disease (LSD). It results from deficiencies in acid β-glucosidase (glucocerebrosidase [GBA], E.C.3.2.1.45). In GD, the accumulation of GBA in macrophages leads to organ dysfunctions. These organ dysfunctions are generally characterized by anemia, thrombocytopenia, bone disease, and hepatosplenomegaly. However, GD is rarely associated with neurological symptoms (1-2). There are three clinical subtypes of GD according to the absence or presence of neurological complications. Type I GD (OMIM 230800) or non-neuronopathic form manifests without neurological involvement. Type II (OMIM 230900) or acute neuronopathic disease is a fatal neurodegenerative disorder of infancy, and finally, type III (OMIM 231000) is a chronic neuronopathic form of the disease (3, 4). 

The *GBA* gene is located on chromosome 1q21. It has a sequence of 7.6 kb, comprising of 11 exons and ten introns (5). Determination of the GD mutation spectrum in different ethnic groups can be useful in genetic counseling, screening programs, and identification of the molecular basis of GD. The present study aimed to identify the spectrum of *GBA* gene mutations in Iranians with GD and to study the genotype/phenotype associations.

## Materials and Methods

**Study population**

A total of 33 GD patients, including the patients’ siblings and other relatives, were identified in this study. Finally, 26 unrelated GD patients were recruited from different regions of Iran. The study sample consisted of 11 males and 15 females in the age range of 2.5 to 33 years. The patients’ characteristics, including major clinical features, are shown in Table 1. The parents were first cousins in 15 families, while in three families, they were distant relatives. Patients were recruited from specialized pediatric clinics of inherited metabolic diseases and digestive disorders at Mofid Children's Hospital and Ali-Asghar Children's Hospital (Tehran, Iran) over two years.

All patients were previously diagnosed with GD, according to a fluorometric assay of GBA activity or had Gaucher cells in the bone marrow. Additionally, a fluorometric assay was performed for measuring the GBA (**6**) and chitotriosidase (**7**) activities in patients. The high-density lipoprotein (HDL)-cholesterol level was also measured after precipitation of lipoproteins containing apo B with phosphotungstic acid, using an available kit (Pars Azmoon Co., Iran). Seventeen patients received enzyme replacement therapy (ERT). This study was approved by the Ethics Committee of Pasteur Institute of Iran (IR.PII.REC.1394.02). Written informed consent was also obtained from the controls, patients, or their parents.

**Genotyping**

Genomic DNA was extracted from peripheral blood leukocytes, based on the standard phenol-chloroform method using a commercial SinaClon kit (Tehran, Iran). First, the samples were screened for common p.N409S and p.L483P mutations by polymerase chain reaction (PCR)-restriction fragment length polymorphism (RFLP), using XhoI and NciI restriction enzymes, respectively. The presence of both mutations was confirmed by DNA sequencing.

For sequencing analysis, eight DNA fragments, which covered 11 exons and exon-intron boundaries of *GBA* gene (NM_001005742.3) and no GBA pseudogene, were amplified by PCR, using exon-specific primer sequences reported in previous studies (8**, **9) (Table 1). On the other hand, the new *GBA*-specific PCR products were prepared by pairing different primers and comparing the sequencing results against both *GBA* gene and pseudogene sequences. Moreover, an ARMS PCR technique was designed for rapid detection of p.S439G mutation, as the third common mutation in our population. The primers were manually designed and evaluated (Table 1). Finally, all PCR products were sequenced at Macrogen Inc. (Seoul, South Korea).

For analyzing and predicting the potential pathogenicity of novel mutations in the *GBA* gene coding region, three web-based tools, that is, SNPs3D (http://www.snps3d.org), SIFT (http://sift.jcvi.org/), and PolyPhen (http://genetics.bwh.harvard.edu/pph2), were used (10**-**12). Finally, in four common new mutations (p.S439G, p.M455R, p.L393V, and p.H312D), mutant structures were constructed using the I-TASSER (13) server, according to the 1OGS native structure.

## Results

A total of 49 *GBA* mutant alleles were identified in 52 chromosomes of 26 GD patients, using PCR-RFLP and direct sequencing of PCR products. No lesions were detected in 30 control alleles. Among mutant alleles, there were 49 single nucleotide missense substitutions (six new mutations and six previously described mutations), one nonsense, and two complex allele mutations.

**Figure 1:**
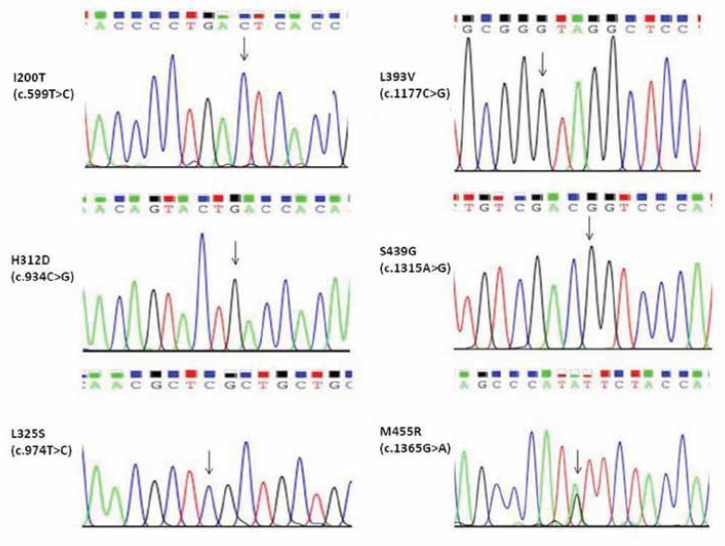
The sequencing results for six new mutations

Mutations found in the present study are demonstrated in Table 2. The mutation results were also confirmed in the DNA samples of the patients’ parents if available. The most commonly observed clinical phenotypes were splenomegaly (84.6%), hepatomegaly (65.3%), anemia (61.5%), thrombocytopenia (61.5%), bone disease (26.9%), and failure to thrive (19.2%), respectively. The sequencing results of the newly detected mutations in the present study are shown in Figure 1. The mean activities of GBA and chitotriosidase were 0.64±0.41 µmol/L/h and 10477±11925 nmol/ml/h, respectively.

The type and frequency of the detected alleles are demonstrated in Table 3. The mutation nomenclature followed the Human Genome Variation Society’s mutation nomenclature recommendations (http://www.hgvs.org/mutnomen). Moreover, the traditional nomenclature, which subtracts the first 39 amino acids of the preprotein, was described (14). The p.L483P mutation was detected as the most common mutation (32.7%) in our patients. The p.N409S mutation was identified in ten alleles (19.2%). The p.S439G mutation was found to be the third most common mutation (7.6% of alleles). This mutation is the first common novel sequence alteration, caused by G to A variation at position 1315 of cDNA (exon 9). The previously described and new mutations of *GBA *gene, nucleotide alterations, amino acid changes, and their frequencies in our study are listed in Table 3.

The new p.M455R mutation was found to be heterozygous in two patients. The second mutant allele was not identified in one of the two patients carrying the p.M455R mutation. In two patients, the combined alleles of R202Q+N227S and S439G+E379K were detected. Nevertheless, in another patient, no mutation in exons or exon-intron boundaries of *GBA *gene was found. The newly detected mutations of *GBA* gene were analyzed *in silico,* using web-based SNP3D, SIFT, and Polyphen2 tools to predict the possible damaging effects of new *GBA* mutations.

## Discussion

The present study is the first report of GD mutations in Iranians with different ethnic backgrounds. The detection rate of mutations was 94.2%, and the p.L483P mutation was the most common mutation with a frequency of 32.7%. Our findings are consistent with several studies on Syrian, Western Indian, Korean, Filipino, and Chinese populations (15**-**19). However, among American, Brazilian, Venezuelan, European, and Jewish populations, the p.N409S mutation was found to be the most common mutation (20**-**24). The prevalence of p.L483P mutation clearly decreased from west to east, while the prevalence of p.N409S mutation increased.

Consistent with other reports (16), a similar phenotype-genotype correlation was found in GD patients with both GD type I and III carriers of p.L483P homozygous genotypes. Interestingly, the most severe involvement was reported in patient 4, who presented with ataxia, history of seizures, cognitive impairment, strabismus, supranuclear gaze palsy (frozen eyes), and other classic symptoms of GD. She also had a history of hypothyroidism and was unable to walk. The second most common mutation was p.N409S, which was found in six patients without any neurological symptoms, indicating the neuroprotective function of this mutation (25).

Moreover, the novel p.S439G mutation was found in the present study. Both patients with this mutation had GD type I with moderate-mild symptoms. However, patient 17 had an unknown type of GD, which co-occurred with beta-thalassemia minor and severe manifestations. She had a moderate cognitive impairment (according to the Mini Mental State Examination [MMSE]), slurred speech, failure to thrive, developmental delay, and learning disorders, but had no other neurological symptoms. Moreover, a patient with the p.L393V mutation was found, who had type I GD. This patient (No. 19) started ERT at the age of three, but suffered from bone pain and was at a high risk of bone fracture, according to the bone mineral density (BMD) test.

In our study, another interesting phenotype-genotype correlation was found in a patient with a homozygous p.D448H mutation. This young female patient had been diagnosed with type III GD at the age of four. She had been on ERT from the age of 14, with a good response to the treatment of anemia and organomegaly. However, at the age of 19, she suffered from oculomotor apraxia and poor eyelid function (decreased blink frequency), which led to a scar in the left eye with keratoconjunctivitis, ocular hypertension, and face asymmetry. She also had a history of kidney stone, migraine, slow speech, and calcification of heart valves, which have been previously reported for this genotype (26).

We identified homozygous p.I200T, p.H312D, and p.L325S mutations in three patients. All of these patients had GD type I with moderate complications and a good response to ERT. The results of *in silico* analysis revealed that all new mutations might be damaging. However, according to all three web-based tools, the p.H312D mutation might be a tolerable alteration in the *GBA* gene. Nonetheless, patient 22 only had this mutation in the *GBA* gene. After starting ERT, organomegaly and anemia improved in this patient after eight months of therapy. The p.I200T and p.M455R mutations were detected with different nucleotide changes in GD (27**, **28). Clearly, these missense changes were pathogenic.

In the present study, the new homozygous p.R202Q+p.N227S mutation was detected in a patient with type I GD. This patient was diagnosed with severe thrombocytopenia and failure to thrive. In Korean patients, the p.N227S mutation (17) was associated with type III GD. In our study, the presence of p.N227S+p.R202Q led to type I GD, without any neurological symptoms.

Concerning the *in silico* results, change of charge in the residues of domain III in the presence of two new p.H312D and p.M455R (α_8_-Heilx) mutations could alter the spatial configuration of GBA molecule, possibly affecting its catalytic function. Histidine 312 and histidine 262 are on the surface of GBA opposite to loop 1 (29). Also, the presence of c.974T>C transition results in the substitution of leucine to serine (p.L325S), i.e., a *non-polar amino acid replaced by *a polar amino acid with a hydroxyl group in domain III. This domain has (β/α) an 8TIM barrel structure (located between α5 helix and β5 sheet); the mutation could change the polarity and affinity for H^+^ with severe phenotypic consequences (30). The p.S439G mutation could eventually disrupt the H-bond between D438 and S439 in loop 2 of GBA in both states of open and close conformations (31).

**Figure 2:**
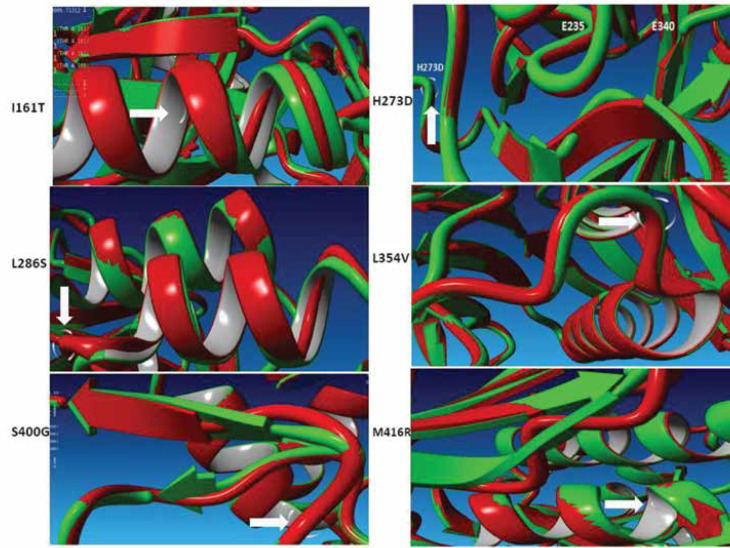
The representation of superposed native GBA structure (green) with different mutant structures (red).

Moreover, the outputs of I-TASSER server were obtained, and all six new mutations were superposed by YASARA structure package (Figure 2). All mutant structures had a root mean square deviation (RMSD) of about 1.2-1.35 Å, which possibly destabilizes the GBA structure. Briefly, in the p.I200T mutation, an extra turn was formed in the α_2_-helical structure. In the p.H312D structure, a part of β_5_-sheet (near the catalytic site) altered to coil. In the p.L325S mutation, Asp322 to Arg324 residues in the α_5_-heilx forms a secondary structure turn. The L354V mutation moderately changed the catalytic site. The p.S439G mutation induced a small β-sheet transition to coil in domain I. Finally, the p.M455R mutation had the same effect on the β-sheet in domain II (Figure 2). The percentage of unknown alleles (5.7%) was similar to reports in European and Asian populations (32**, **33). 

Despite the availability of detailed and efficient methods for the detection of *GBA* gene mutations, many mutations are still unknown. In summary, the current study investigated the molecular basis of GD among Iranians. Six new mutations were detected among GD patients, and the presence of two common mutations, that is, p.L483P and p.N409S, was confirmed in the Iranian population. Our study provided valuable knowledge about the molecular basis of GD, genotype-phenotype correlations, and allelic heterogeneity of this disease.

**Table 1:** Primer sequences and product sizes for identification of specific *GBA* exons, exon–intron boundaries, and common mutations

Exon or common mutation	Primer sequence (5’-3’)	Restriction enzyme	Product size (bp)	Annealing temperature (°C)
Exon 1	F ATCCTCTGGGATTTAGGAGC R CTGGATTCAAAGAGAGTCTG	-	482	56
Exon 2	F GTCCTAATGAATGTGGGAGACC R AAGCTGAAGCAAGAGAATCG	-	478	60.5
Exon 3-4	F GTTCAGTCTCTCCTAGCAGATG R GCAGAGTGAGATTCTGCCTC	-	637	60.5
Exon 5-6	F GATAAGCAGAGTCCCATACTCTC R ACAGATCAGCATGGCTAAAT	-	644	56
Exon 7	F CTAATGGCTGAACCGGATG R ATAGTTGGGTAGAGAAATCG	-	1144	56
Exon 8	F CTAGTTGCATTCTTCCCGTC R GCTTCTGTCAGTCTTTGGTG	-	404	60.5
Exon 9	F TGTGCAAGGTCCAGGATCAG R GCTCCCTCGTGGTGTAGAGT	-	914	62
Exon 10-11	F ACTGGAACCTTGCCCTGAAC R CTCTTTAGTCACAGACAGCG	-	904	56
N409S (c.1226A>G)	F GTCTCTTTGCCTTTGTCCTTACCCTCGA R ACTGTCGACAAAGTTACGCACCCAAT	XhoI	120	60.5
L483P (c.1448T>C)	F ACTGGAACCTTGCCCTGAAC R CTCTTTAGTCACAGACAGCG	NciI	904	56
S439G (c.1315A>G)	F AGCTGCCTCTCCCACATGTGACCCTTAC (common) R GTCCTTGGTGATGTCTACAATGATGG**A**ACT (wild) R GTCCTTGGTGATGTCTACAATGATGG**A**ACC (mutant)	-	210	61

**Table 2:** Demographic data, clinical characteristics, and geographic area of Iranians with GD

No	Sex	Age (y)	Genotype	Phenotype	GD Type	Age at diagnosis (y)	CHIT^1^	HDL^2^	Cons^3^	Ethnicity^4^
1	M	9.5	L483P/L483P	At^5^, BD, FT, S, SNP, T	3	1	2381	22	+	Fars
2	M	4	L483P/L483P	A, H, Partial Sp	1	3	11574	23	+	Kurd
3	F	7.5	L483P/L483P	A, HS,OH, SS, St, T	3	3	38131	32	+	Fars
4	F	3.5	L483P/L483P	A, At, CI, DD, Hsz, St, S, SNP, T	3	2	6193	25	+	Fars
5	F	8	L483P/L483P	A, HS, T	1	3	14250	16	+	Fars
6	F	7	L483P/L483P	A, BD, FT	1	2	1600	28	-	Lor
7	M	10	L483P/L483P	A, HS, N, T	1	1	3483	45	-	Gilaki
8	M	9	L483P/H312D^6^	A, HS	1	8	38244	11	-	Azari
9	F	15	L483P/N409S	A, BD, Sp	1	8	1801	26	-	Fars
10	M	4	L483P/M455R	HS, T	1	3	3015	32	-	Fars
11	F	25	N409S/N409S	H, Sp	1	23	1450	35	+	Fars
12	M	33	N409S/N409S	C, S, T	1	31	3411	24	+	Gilaki
13	F	11	N409S/N409S	HS	1	6	4527	29	+	Azari
14	M	29	N409S/N409S	A, HS, T	1	14	5714	28	-	Azari
15	F	10	N409S/W420X	HS	1	7.5	6670	36	-	Gilaki
16	M	9	S439G/S439G	A, BD, HS, T	1	3	27406	12	+	Kurd
17	F	7	[S439G/S439G + E379K]	A, CI, DD, FT, HS	?	3	8004	18	-	Kurd
18	F	29	M455R/?	A, BD, HS	1	22	11577	19	+	Azari
19	F	9	L393V/L393V	BD, S, T	1	3	3491	10	+	Azari
20	F	19	I200T/I200T	S, T	1	15	101	33	+	Kurd
21	M	7.5	[R202Q/R202Q +N227S/N227S]	FT, S, T	1	6.5	2947	30	+	Azari
22	M	2	H312D/H312D	A, HS	1	1.5	36731	18	+	Lor
23	M	3	L325S/L325S	A, FT, HS, T	1	0.5	0	19	+	Gilaki
24	F	2.5	R398Q/R398Q	A, HS, T	1	2	21647	19	+	Fars
25	F	18	D448H/D448H	BD, OH, OMA, S, SS, T	3	4	12390	24	+	Fars
26	F	20	?/?	A, C, HS, T	1	19	5677	30	+	Gilaki

^1^CHIT: Chitotriosidase activity. ^2^HDL-C: HDL-cholesterol. ^3^Cons: Consanguinity. ^4^Main ethnicities in Iran include Fars, Azari, Kurd, Gilaki, and Lor, respectively.

^5^Abbreviations: F, female. M, male. y, year. A, anemia. At, ataxia. BD, bone disease. C, cirrhosis. CI, cognitive impairment. DD, development delay. FT, failure to thrive. H, hepatomegaly. Hsz, history of seizure. MC, microcephaly. N, nocturia. OH, ocular hypertension. OMA, oculomotor apraxia. S, splenomegaly. SNP, supranuclear gaze palsy. SS, slow speech. Sp, splenectomy. St, strabismus. T, thrombocytopenia

**Table 3:** Prevalence of various *GBA* mutant alleles, cDNA nucleotide substitutions, amino acid changes, and frequency of mutations

Allele type^a^	Exon number	cDNA^b^	Protein	Frequency (%)
Missense L483P N409S S439G M455R L393V H312D I200T L325S R398Q D448H	10 9 9 9 8 7 6 7 8 10	c.1448T>C c.1226A>G c.1315A>G c.1365G>A c.1177C>G c.934C>G c.599T>C c.974T>C c.1193G>A c.1342G>C	p.Leu483Pro p.Asn409Ser p.Ser439Gly p.Met455Arg p.Leu383Val p.His312Asp p.Ile200Thr p.Leu325Ser p.Arg398Gln p.Asp448His	17 (32.7) 10 (19.2) 3 (5.7) 2 (3.8) 2 (3.8) 3 (5.7) 2 (3.8) 2 (3.8) 2 (3.8) 2 (3.8)
Nonsense W420X	9	c.1259G>A	p.Trp420X	1 (1.9)
Complex allele R202Q + N227S S439G + E379K	6 & 6 9 & 8	c.605G>A c.680A>G c.1315A>G c.1135G>A	p.Arg202Gln p.Asn225Ser p.Ser439Gly p.Glu379Lys	2 (3.8) 1 (1.9)
Unidentified allele	-	-	-	3 (5.7)
Total				52 (100)

^a^ *GBA* mutations are named according to www.hgvs.org/mutnomen.

^b^Nucleotides are numbered from A of the first ATG.
